# Rip Van Winkle, Dentist

**Published:** 1879-07

**Authors:** W. H. Robinson

**Affiliations:** Suison, Cal.


### RIP VAN WINKLE, DENTIST.

BY W. H. ROBINSON, SUISON, CAL.

"A little nonsense now and then,
Is said to be good for the best of men."
When I say you are all wise men
Modesty may prevent a reply,
But that this is nonsense,
I know you will all vote "aye."
You have all heard of Rip Van Winkle, a sleepy old chap,
Who took at one time a wonderful nap
Of twenty years' duration. But a little mishap

Disturbed his repose, so he woke up and looked around,
And such strange and wonderful changes found
That he thought his body was under the ground,
And his spirit had wandered off in space,
He scarcely knew a person or place.
But we must leave old Rip alone in his glory,
While about a new Rip I tell you a story.
And he was a dentist, that is, his sign read so,
Or had, so old folks said, twenty years ago.
Our hero was born in the dark of the moon,
And very late in the afternoon,
And, we think, slightly out of tune
With the present generation.
Over his face he had two veils,
A sign that midwifes say never fails,
To show that the wearer will be good and great,
Unless, like Rip, they are born so late,
And go so slow that it would take fortune or fate
A thousand years to make them so,
And, like modern belles, they don't like to wait
So long, and then have to make the man,
When plenty ready made await their hand.
Rip ate and grew in the usual way,
And in due time became a man,
And his course through life he began
To consider, map out and plan,
And the future to carefully scan,
For some comfortable retreat;.
Where he could get his bread and meat,
Without too much labor of body or mind,
To neither of which was Rip much inclined,
His usual place being far behind,
And the world before him. Out he must go
To begin life's battle, to hoe his own row,
But where or how to begin he did not know.
His cash on hand was a very small pile,
And his coat wasn't cut in the latest style,
No. 18 brogans and a hickory shirt.

Rip never had any great aversion to dirt.
He fitted his pants like a knife its sheath,
And never gave thought or brush to his teeth.
About this time Rip had a new sensation,
That caused him considerable consternation,
And gave him "right smart" of pain and vexation.
Rip thought it worse than a small earthquake,
From his crown to sole it made him shake,
A big molar was "holler," and didn't it ache?
Rip made a bee line for the old M. D.,
And he for Rip's tooth with his old turnkey,
And missed it. The tooth didn't come out.
Didn't Rip yell, and roar, and shout
Murder? Doctor one round is enough for me,
While the Doctor contended for two or three,
And swore he knew what he was about,
And would break his key or have that tooth out.
So he tugged and twisted till with a crash and a roar
Rip and the Doctor rolled on the floor.
As soon as the smoke of the battle was o'er,
Rip found the key broken and he was glad,
But the tooth was still in and the Doctor was mad,
And so was Rip, for the tooth was there;
And didn't it ache? It just made his hair
Stand on end. Rip had been taught not to swear,
And yet there sounded through the air
Words very unlike his evening prayer.
Rip was mad, so mad, I know of nothing to compare
With him, mad as a hornet, tiger or bear,
Or as some folks say, as mad as a March hare,
However, this is a purely empirical simile,
For I know not what a March hare is, or how mad it can be.
Rip was mad as a dentist when he works hard all day,
And then gets bilked out of his pay,
As returns the jockey when beaten in the race,
Or the tired hound when worsted in the chase,
Or the truant school boy flogged and in disgrace.
So Rip went home, mad and blue,

And that tooth aching, whew!
He just did not know what to do.
All known remedies that tooth defied,
And ached worse and worse the more he tried.
The neighbors came in and each had a way
To cure that tooth without delay,
Till the doctoring Rip had to endure
Was worse than the ache they wanted to cure.
Paregoric, pain killer, capsicum,
Camphor, cloves, pepper and rum,
Salaratus, ginger, turpentine, spice,
Poultices, hot as fire and cold as ice,
Till Rip thought he must freeze or boil,
And then came smoke, snake and wizzard oil,
And things that never were known to fail,
Such as spider's web and rattlesnake tail,
But none of these were of any avail.
So that tooth ached on and kept Rip howling,
Till a neighbor announced there was that prowling
About a man who stuffed teeth with gold and tin,
Pulled old ones out and put new ones in,
And could cure a tooth no matter how sick,
And do it up on the double quick.
When Rip heard this, like the hare from the hound,
He made rapid strides till the tooth man he found
On the road with his office—for he carried it 'round.
A log by the fence made a strong dental chair,
And the tooth man brought forth a wonderful pair
Of "pullikens" long, strong, crooked and stout,
And went for that tooth and soon had it out.
And Rip was jubilant, his pain was over,
He felt as gay as a pig in clover,
Till the tooth man said, "the fee I gits
"For such jobs as this is always four bits."
This placed Rip in awkward stew,
For all the money he had was two.
Said Rip, "for four bits I work a whole day,
"Four bits for one minute is awful big pay."

Said the tooth man, "two bits is the doctors' charge;
"I am a dentist, sir, my fee is not too large."
"A what?" said Rip, as he opened his eyes,
And viewed the tooth man from head to toe.
"A *dentist*, sir, I would have you know."
"A dentist?" said Rip, "I don't know what that be,
"I thought you a tooth puller by the way you served me.
"I for four bits do a whole day's work,
"And a dentist is one that gets four for a jerk.
"If a dentist can so easily make such a fee
"What will you take and make a dentist of me.
"I would like to learn a trade
"At which money is so easily made."
The dentist and Rip soon made a contract,
By which he agreed to learn Rip to extract
Teeth and roots of every kind and description,
And give him a royal secret prescription
To cure all their aches and all their holes stuff,
And start him in biz with tools enough
To place him away up in the dental profession.
A month was the time for Rip's education
And twenty-five dollars the remuneration.
Rip was educated on a high pressure rule—
We all know it must be a wonderful school
That can make either a wise man or fool
For twenty-five dollars in thirty days.
But in that time Rip crammed his pate—
In a way he was sure would make him great—
With dental mysteries, science and arts
He was sure he was master of all its parts,
And had become so wonderful wise,
He knew all that was worth knowing under the skies,
And that his head held more dental lore,
Than ever was held by a head before,
He was afraid it would bust if he learned any more.
And presto! in just one month to a day,
Dr. Rip Van Winkle, Dentist, ever so gay,
Steps forth, fully prepared, in every way,

To stuff teeth, and put in and take out.
And make all plugs so good and stout,
And fully warranted in every way
To last at least one year and a day.
So Dr. Rip peddled his trade as he had been taught,
And from house to house business sought,
And many were the victims he caught.
Merciful God, hear our prayer
For the victims who came to Dr. Rip's chair
To have their molars put in repair,
For if they were not in hell while there
There is no hell anywhere.
Of tramping about Rip soon got tired,
And then a professional office he hired,
And a large sign in yellow and blue,
Told what Dr. Rip Van Winkle could do
In making *old teeth* better than new.
No, I have got that wrong end too.
Making new teeth better than old,
Was what that sign must have told.
Old or new, new or old, what a bother,
I have got that mixed, 'twas one or t'other.
Rip knew which, for he verily thought
That he so much wisdom had bought
That if the world treated him as it ought
He soon to fortune and fame would be brought.
So he stuffed and pulled from day to day,
Always keeping an eye on the pay,
And soon had some coin laid away,
And then he began to show his oats
In the gloss of his hats and the cut of his coats;
And to put on lots of professional style,
So that in a very short while
He thought Dr. Rip a professional sun
To whom other dentists for light must come.
But they didn't. They let him alone.
So in one small room his great light shone,
And with this grand illumination

We will take a look at the situation.
He had a chair, and a stout one, too,
His grandfather remembered when it was new.
His spittoon—a drygoods box full of sawdust,
A few ancient forceps covered with rust,
Files and burs decidedly smooth,
Not rough, like common dentists' use.
Excavators and pluggers quite a lot,
When they were new Rip had forgot.
New things to Rip were an innovation;
In that way he had no inclination.
His laboratory—I pass it by,
I couldn't describe it if I should try;
But everything was good and old.
Rip always did as he had been told
By his illustrious preceptor.
Rip's sanctum was a wonderful pen,
Or shop, or office or dental den,
Or whatever you wish to call it.
But he plugged and stuffed from year to year
Without the least doubt or fear,
But that the highest place he had gained
To which a dentist had ever attained.
So he shut himself up in his dental pen
And looked with disgust on those silly men,
Who in professional journals tell
Of the failures they make and what they do well,
And in associations are wont
To talk of the little they know and the much that they don't;
And about abscess and nerves fine theories spin
And what foil, cohesive, soft, thick or thin,
Is best to fill teeth, and how to begin
What they call "contour restorations."
To all these new fangled innovations
Rip was as deaf as an adder is said to be—
That an adder is deaf I doubt, but use the simile,
As Rip used many things in his profession,
Taking them on hearsay, and asking no question

About absurd things he heard in his youth,
But of all new facts in science, doubting the truth.
So Rip stood still and let the world go
As he could neither stop it or make it move slow.
But to all progress in dentistry Rip often tried
To have some old departure brake applied,
As onward and upward and forward it hied,
Saving empirical paths for the great highway
Of science and truth which it made pay
Tribute to its genius, as from day to day
It gathered fresh wreaths from the storehouse of truth.
On its banner "excelsior," just like the youth
Who wrote that for his mother and placed it up high
With a manly resolve to reach it or die.
So the dental profession no brake could hold back
As onward and upward it made its track,
Scarcely pausing to hear the quack
Say it was going too fast, if it went any faster
It would "burst its boiler." And such a disaster
Fogies said the profession was fast approaching.
On its collateral mother so enroaching,
They very much feared a monster abortion,
That would mix D. D. S. and M. D. in such contortion,
That no one could tell the child from its mother,
Or know "t'other from which, or which from t'other."
But the dental profession advanced every year,
Leaving fogies and failures far in the rear,
So far that naught but their wails it could hear,
As they sighed for the days of long ago,
When the dental profession used to go slow.
During all these changes in the world and men,
Rip kept himself close in his dental pen—
His countenance grave, his brain rather mazy—
Declaring the dentists had all gone crazy.
Rip took no journal, read few books,
But through some odd niches and nooks,
Ideas would creep into his head
That filled him with a feverish dread.

Even the daily papers he read
Told things about dentists that made him scowl,
Shake his head and look wise as an owl.
But as from month to month and year to year
So many things did appear,
That Rip got sadly out of gear,
He believed in nerve killing but not extirpation,
And the good old plugger without any serration.
But when he heard of nerve capping and curing abscess,
Rip was in such mental distress.
But when he heard of mallets, hand and pneumatic,
And pluggers, electric and automatic,
And that the profession's approval of these was emphatic,
Poor Rip, as you may well suppose,
Become bewildered and comatose,
And his mental condition became so hazy,
He knew not whether he or the profession was crazy.
And when there followed in quick succession,
So many new things into the profession,
At engines and discs he was soon amazed,
At rubber dam completely crazed.
So he shut himself up in his dental pen,
Vowing he would not go out again.
But like the bear in the hollow tree,
He would incubate till the profession was free
From such tomfoolery, and back where it used to be.
So Rip did like his grand dad, who, you know,
Took a nice little nap for twenty years or so,
And went into a state of somatic incubation,
Of he never knew how long duration.
But it seemed very long and very profound,
For one that was not under the ground.
Rip was happy as happy could be,
From engines and dams he was free.
Dr. Rip Van Winkle, "Requiescat in pace."
The world went on, turning o'er and o'er
Not even stopping to hear Rip snore.
And the dental profession went jogging along,

Something new was its daily song,
Indeed, new things made it strong.
Ye who really want to know
How fast the profession is wont to go,
Come back with me for twenty years.
How different everything appears,
No mallets, no discs, no engine, no dam,
All these, done now, was then done by hand.
A thousand things that now the dentist assist,
He got along without—they did not exist.
Yet to-day many teeth can show
Beautiful fillings done twenty years ago.
Monuments to hands that are now at rest,
May we, like them, do our level best,
On every tooth we try to save,
So that when we are laid in the grave,
We'll leave golden monuments to tell,
The work our hands did, and that they did it well.
"Tempus fugit" the world rolls round,
Rip has been snoozing very profound;
But now he begins to hear a sound,
Not very loud, but strange and queer
Is the buzzing and rattle that reaches his ear,
Which Rip has a strong disinclination to hear.
Still the buzzing goes on, and the rap, rap, rap,
And Rip is disturbed in his wonderful nap;
And as the buzzing and rapping louder grows,
Rip can not enjoy his sweet repose.
So he rubbed his eyes and seemed to awake,
And looks around. What makes him shake
And tremble in fright and amaze?
What wond'rous things meet his gaze?
It must be the light of other days.
Over his window, in plain view,
Hangs a sign that looks rather new,
And on it Rip Van Winkle, D. D. S.,
But what it meant Rip couldn't guess.
Instead of his pen a beautiful room,

And a lady as fair as a rose in bloom,
Sat in a beautiful fairy-like throne,
So beautiful that it fairly shone
Like a crown of gold or jewels rare.
It moved up and down, forward and back,
It had more joints than a jumping-jack.
And by the side of the lady and throne,
Stood a man that Rip thought in other days he had known.
His hand was guiding a strange machine—
The strangest Rip had ever seen—
And it was making that buzzing sound
That roused Rip from his nap profound.
Over the lady's mouth was a very strange veil,
And from that machine came a long, slender tail
That looked like a snake on its end was a sting,
Or at least a funny, rattling thing.
And it went buzz and rap, rap,
Rip knew it was what had disturbed his nap,
And as he drew nearer for close inspection,
The lady seemed to be taking an oral injection,
That, in a strange and magical way,
Made golden crowns, all shiny and bright,
Grow on old roots. 'Twas a beautiful sight
To see the eruption of a nice golden tooth,
And made Rip's heart beat with the warm glow of youth.
As when the chrysalis warmed by the spring,
Goes forth a butterfly on bright golden wing,
Or, as the spirit from the body set free,
The glories of heaven is permitted to see,
Is bewildered, enraptured, entranced with delight,
So was Rip with this beautiful sight.
In utter amazement it made him stare,
Like a porcupine's quills stuck out his hair,
As he gasped, "Am I in heaven, or hell, or where."
But the fairy queen, from her beautiful throne,
With a musical voice and a mouth that shone
With crowns of gold, said, fear not Rip,
You are alive and well,

And still on the earth where molars dwell.
Look at my teeth, this throne and machine,
And tell me if the like you ever have seen,
And how you would like that dentist to be,
And with these nice machines fill teeth for me.
"Splendid," said Rip, for such a mutation
Would give me a wonderful reputation;
But ain't the profession going too fast,
Do solid gold crowns last
Like the good old plugs of amalgam and tin,
That my preceptor used to put in?
Look, said the queen, at that D. D. S..
Try if his name you can guess,
Oh Heavens! said he, do my senses lie?
Can I be that man and that man be I?
Can these things be true? and the queen said yes,
You are now Rip Van Winkle, D. D. S.,
You have been off to the dental college
Where you got a little knowledge,
Just enough to show to you
The kind of work other dentists do,
And how much you may learn
From the profession you now spurn.
These things are real that you now view
And just what you ought to be and do.
Rip began to realize they were true
And felt himself thrilled with a strange delight
And quite enraptured with the beautiful sight,
So many instruments new and bright,
That beautiful queen on her fairy throne,
And her fine golden crowns Rip's own
Operations and Rip D. D. S. to a queen,
And now for the first time he tries that strange machine,
Just to finish a nice golden crown,
When lo! he hears that buzzing sound,
That wakes him from his sleep profound,
Rip rubs his eyes and looks around.
Presto, and everything is changed;

Rip is sure he is now deranged.
But, as memory backward sped,
And Rip felt his feet, body and head
And realized that he wasn't dead.
He found himself in his dental pen
And everything as it always had been.
That day Rip had read a free dental journal
Full of cuts of machines. Rip called them infernal
And only fit for those who were vernal,
But such strange ideas into his head did creep
That Rip fell into a wonderful sleep,
As he studied them o'er in his old dental chair,
And his professional digestion being out of repair.
He was somewhat troubled with the nightmare.
As he was enjoying that wonderful nap
From which he was aroused by that strange, buzzing rap,
From that beautiful vision, so real did it seem.
"Sic transit gloria mundi," 'twas but a dream.

MORAL:

All the Rips are not dead yet.

